# Research on instability characteristics and precursory effect of coal-rock parting-coal structures

**DOI:** 10.1038/s41598-022-15738-x

**Published:** 2022-07-15

**Authors:** Yang Liu, Jian-Hua Wang, Cai-Ping Lu, Chao Wang, Hua-Dong Xie, Xian-Yang Yan

**Affiliations:** 1 Key Laboratory of Deep Coal Resource Mining (Ministry of Education), School of Mines, China University of Mining and Technology, Xuzhou, 221116 Jiangsu People’s Republic of China; 2 Xiaoyun Coal Mine, Jining Energy Group, Jining, 272000 Shandong People’s Republic of China; 3 Centre of Rockburst Prevention Research, Shandong Energy Group, Zoucheng, 273500 Shandong People’s Republic of China; 4 Dongtan Coal Mine, Shandong Energy Group, Zoucheng, 273513 Shandong People’s Republic of China

**Keywords:** Civil engineering, Geology, Geophysics

## Abstract

The slip and instability mechanisms of coal-rock parting-coal structures under uniaxial loading conditions were investigated using experiments and case verification. The slip and the corresponding precursors were described by monitoring the displacement, strain, and acoustic emissions (AEs) of coal and rock parting blocks during testing, and the experimental results were verified by analyzing the microseismic (MS) effects during the working face advancing in a coal seam bifurcation area. The main conclusions were as follows: (1) each slip of the discontinuities sandwiched between coal and rock parting produced shear and tensile cracks, but the shear cracks was dominant; (2) for the instability mode that was characterized by low peak stress, high energy release, and a stable *b* value of AE, each slip corresponds to a peak frequency of AE, which can reveal the final instability mode; (3) the sudden drop in the fault total area of AE can be regarded as a precursor for the warning fracture or slip instability of a discontinuity; and (4) the MS events in the coal seam bifurcation area were mainly characterized by a wide frequency and high amplitude, especially near the coal bifurcation line, where there were obvious characteristics of low-frequency shear fracture for the MS events. This study is relevant for the early warning of coal-rock dynamic disasters triggered by the slip, fracture, and instability of coal-rock parting compound structures in coal mines.

## Introduction

Owing to their instantaneous and violent characteristics, coal or rock dynamic disasters seriously threaten the safety of underground engineering structures such as coal mines. Especially in recent years, with the increase in the depth and geological complexity of coal mining, coal or rock dynamic phenomena (e.g., rockbursts) have become one of the main disasters threatening mine safety^1–3^. In addition to the material properties of coal and rock, the geomechanical environment is also an important factor influencing the dynamic instability of coal and rock. Among them, geologically weak planes, such as faults or coal seam bifurcations, are the direct cause of local stress anomaly, failure, and instability in the stope^4–8^. Formation of coal seam bifurcation is related to the crustal movement, geographical environment, and plant accumulation in coal formation period, and it generally appears as that a single coal seam is gradually divided into two coal seams along transverse direction. And near the coal seam bifurcation line, the coal seam presents a combined structure of upper layer of coal seam, wedge-shaped rock parting and lower layer of coal seam from top to bottom. On the one hand, weakness changes the stress distribution of the stope, which can readily cause local stress concentration. On the other hand, the occurrence of the weakness also changes the stability of the coal or rock, which is more likely to induce the deformation and instability of the coal/rock^9–11^. In the coal seam bifurcation area, the petrographical structure is more complex, and the nonparallel discontinuities are prone to instability under the disturbance of excavation, which is a major hazard causing structural instability in the stope. Several rockburst accidents have been proved to be related to the coal seam bifurcation structures^7, 12^.

Early research on the instability of coal/rock weak planes mainly focused on the field of seismology and proposed the seismic mechanism of fault stick–slip^13, 14^, which stimulated extensive research on weakness sliding instability^15–17^. Meanwhile, the weak plane instability theory was introduced in the mining field to explain fault activation and its induced mechanism of acting on rockburst in stopes^18, 19^. In laboratory settings, research on the slip instability of weakness was mostly conducted using the shear slip test of two- or three-body coal/rock compound structures, and the occurrence of shear slip along discontinuities was proved to be related to the shear stress and lateral stress on it, based on the mechanical conditions of the structural instability^7, 20, 21^. At the same time, the local effect of sliding was proposed, considering the heterogeneity of the weak plane^22–24^. In addition, as per the factors influencing slip instability, a large number of research results have pointed to the roughness, stress conditions, and slip weakening behavior of the weak plane^25–27^. Considering the failure and instability of compound structures with parallel discontinuities, most researchers believe that the interaction between coal and rock mass caused by a difference in physical and mechanical properties is the main controlling factor for the instability of the compound structure, which is related to the difference in the elastic modulus and strength of coal and rock mass^28, 29^. Apart from simple shear slip or fracture instability, there are two nonparallel discontinuities of the composite structure of coal and rock in the bifurcation area; thus, it is easier to produce higher shear stress on the discontinuity between coal and rock parting under the action of mining disturbance, which can induce shear slip. In addition, the difference in physical and mechanical properties between coal and rock parting cannot be ignored. Therefore, shear slip along discontinuities accompanied by fracturing of coal/rock masses may be the main form of instability in the compound structure in the bifurcation area.

In the process of deformation and fracture, the loaded coal/rock will produce a certain acoustic/seismic response, such as acoustic emission (AE) and microseismic (MS) signals, which is an important means to track its crack propagation, deformation, and fracture characteristics^30, 31^. Based on the vibration waveforms monitored by AE and MS systems during the deformation and fracture process of coal and rock, basic information such as the location, frequency, and energy of cracks can be obtained directly^32–35^. Furthermore, the AE/MS characteristics throughout the deformation process, fracture, and instability of coal/rock can be further analyzed to reflect their evolution characteristics. With the progress of data analysis technology, AE/MS data have been gradually used in the tomographical studies of stress distribution^36–38^, solutions for focal mechanisms^39, 40^, and analyses of precursor effects of macroscopic failure^41, 42^. In terms of precursory effect analysis, the *b* value and fault total area (FTA) of AE/MS events are widely used, and the accuracy of the prediction results of these parameters has also been recognized by many scholars. A large number of studies have shown that the *b* value of vibration events can reflect the scale of crack propagation, in which high *b* values are generally related to the development of microfractures, whereas a decrease in *b* value indicates that the microfracture expands and penetrates to form macrofractures^43–45^. FTA represents the fracture scale of coal/rock based on the occurrence frequency and energy of vibration events, which can overcome the defects of the representation mode with a single parameter. Among them, an increase in FTA indicates the rapid development and expansion of cracks, which is also a precursor signal of macrofractures^46^.

Affected by its wedge-shaped rock parting, the physical and mechanical structure of the three-body compound structure of coal and rock parting is relatively complex, and both shear slip along the inclined discontinuities and torsional deformation of the coal/rock will occur during the process of deformation and instability of the compound structure, which will result in the failure and instability of the structure characterized by shear slip accompanied with fracture. Therefore, the failure and instability characteristics of the coal-rock parting compound structure is more complex than the single slip/fracture and instability, and the identification of precursory information will also be more difficult.

Based on laboratory experiment, this study investigated the deformation, fracture, and instability characteristics of a coal-rock parting compound structure in a bifurcation area—including deformation, crack development, and energy release—and the precursor information of failure and instability was also put forward based on AE signals. In addition, the experimental results were verified by field investigations.

## Experimental investigation of compound structure slip and fracture

### Test apparatus

**Figure 1:**
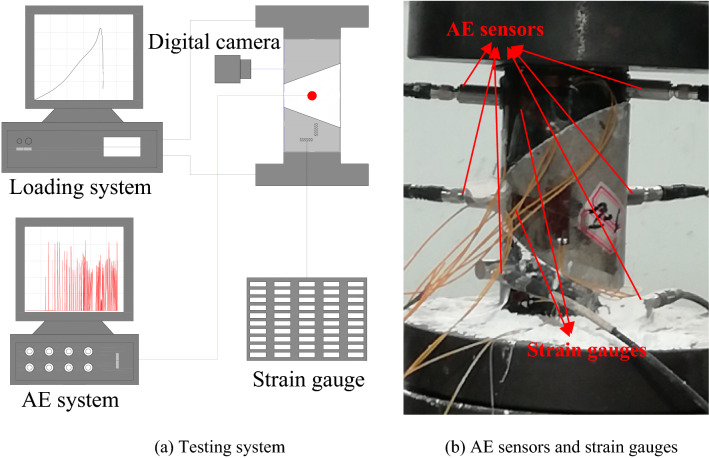
Testing system, acoustic emission (AE) sensors, and strain gauges.

**Figure 2:**
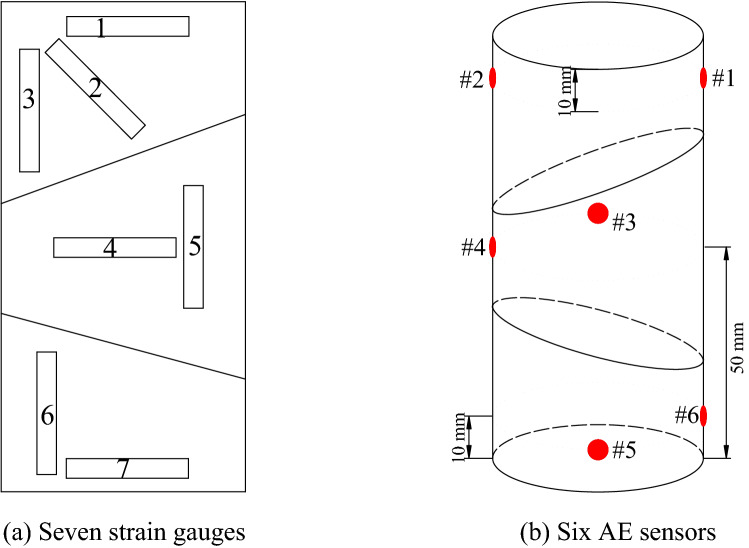
Arrangements of AE sensors and strain gauges.

The experimental setup included a uniaxial loading system, a DH3818Y static strain gauge, a PCI-2 AE system, and a GX-1/3 high-speed digital camera, as shown in Fig. 1a. The AE sensors and strain gauges on the compound samples are shown in Fig. 1b. A total of six Nano30 AE sensors and seven strain gauges were used in the tests, and the three-dimensional positioning of the AE events during slip and fracture of coal-rock parting-coal structures (CRCS) was spatially located. The arrangements of the AE sensors and strain gauges are shown in Fig. 2.

### Samples

Coal and rock parting blocks collected from the Juye mining area were cut into cylinders with diameters of 50 mm and lengths of 100 mm. A total of four compound samples were designed, including upper coal, central rock, and bottom coal, by cutting the cylinders according to different angles of two discontinuities, as shown in Table 1. The upper and bottom end faces of the compound samples were ground until both the overall unevenness and nonperpendicularity were less than 0.02 mm. The uniaxial compressive strength of the coal and rock parting were 20 and 55 MPa, respectively.

**Table 1:** Dip angles of the upper and bottom discontinuities.

No. of CRCS	*α*/°	*β*/°
#1	20	15
#2	25	20
#3	30	25
#4	35	30

### Parameters setup

Uniaxial compression tests were conducted on four samples to investigate the slip, fracture, and instability characteristics of the discontinuities with different contact angles. The displacement control mode was adopted during each test, and the loading rate was set to 0.3 mm/s. Before each test, the strain gauges and AE sensors were attached on the surface of the coal and rock parting, and dry granular quartz powder with an initial particle size of 23 μm and a thickness of 1 mm was evenly spread on the contact surfaces to simulate the structural surface features. The strain gauge, AE system, and high-speed digital camera were employed to monitor and record the process of deformation, slip, fracture, and instability in real time.

### Results and analysis

#### Characteristics of slip and fracture

**Figure 3:**
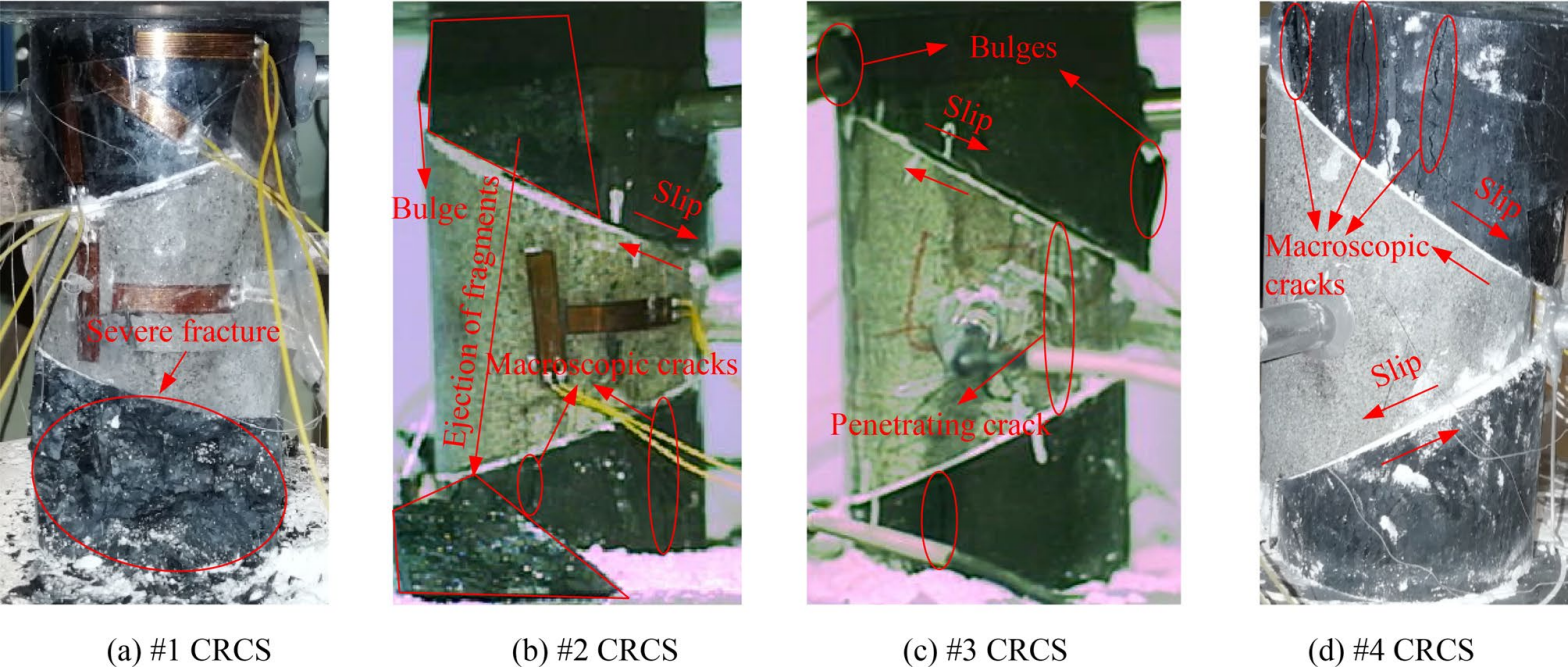
Final instability and failure photos of tested samples.

**Figure 4:**
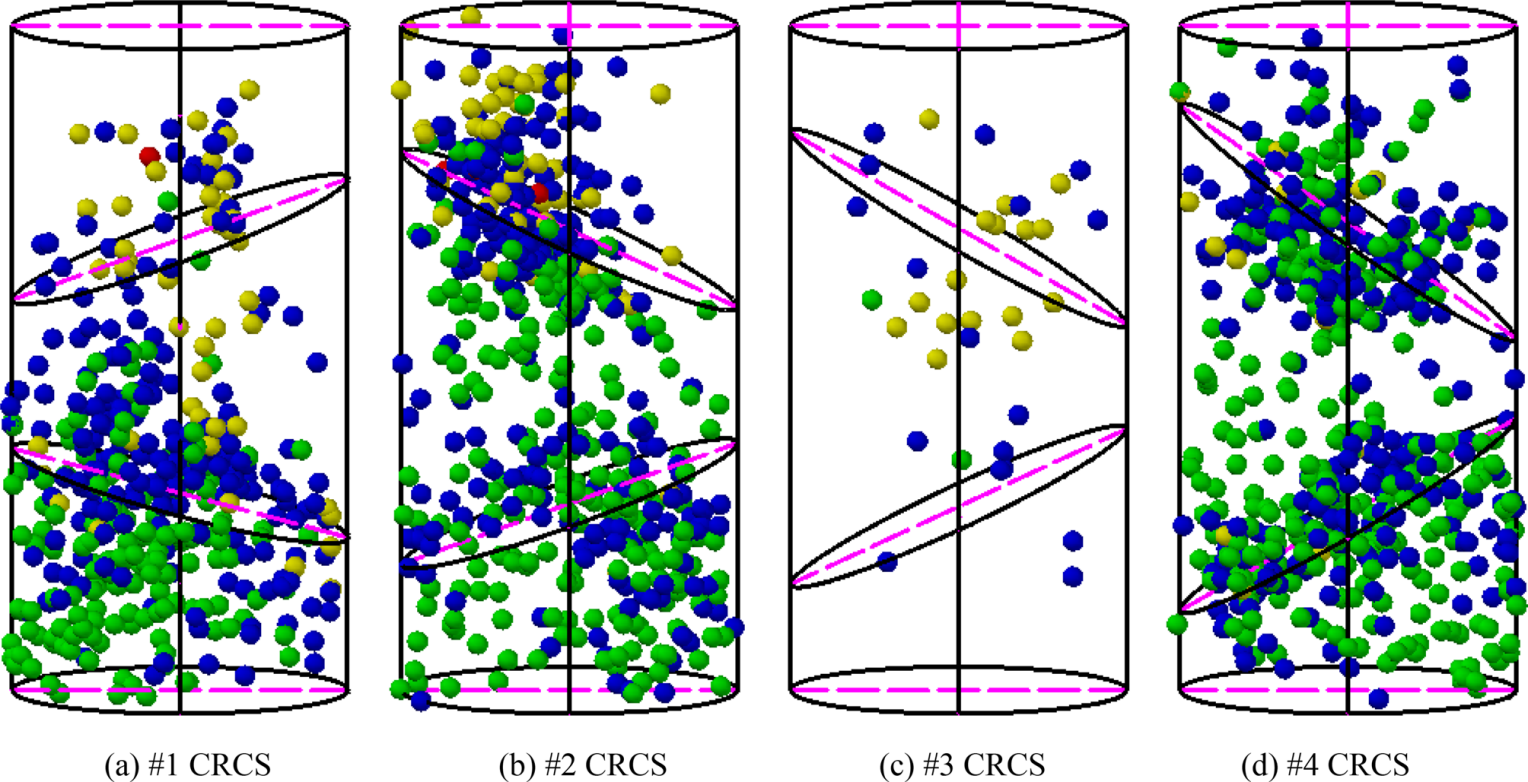
Locations of AE sources during slip, fracture, and instability of the four compound samples. Note: The color of the AE sources denotes energy release (green: 0–10 ms·mV; blue: 10–10^2^ ms·mV; yellow: 10^2^–10^3^ ms·mV; red: 10^3^–10^4^ ms·mV), and the AE energy is time integral of the absolute signal voltage.

The angles of discontinuities of the four samples gradually increased from #1 to #4 (the upper discontinuity was from 20° to 35°, and the bottom discontinuity was from 15° to 30°). Therefore, the effect of discontinuity angle on the characteristics of slip, fracture, and instability can be explored. Figure 3 shows the final instability and failure modes of the tested samples. The locations of the AE sources during slip, fracture, and instability are shown in Fig. 4.

The final instability of sample #1 was mainly due to the failure of the bottom coal block. During the entire loading process, both the upper and bottom discontinuities did not slip significantly (Fig. 3a). The AE sources were mainly concentrated in the rock parting and lower coal block, and some high-energy sources appeared along the discontinuities, indicating significant fracture (Fig. 4a). At the final loading stage of sample #2, spalling of the upper coal occurred, corresponding to the two highest energy sources marked by red balls. Meanwhile, the upper discontinuity produced significant slip, resulting in the final instability (Fig. 3b). A large number of sources gathered near the upper discontinuity, and low-energy sources obviously dispersed in the rock parting and bottom coal block (Fig. 4b). The upper discontinuity of sample #3 produced multiple discontinuous slips, accompanied by the propagation and convergence of macroscopic cracks. In addition, the bottom discontinuity also generated slight slip (Fig. 3c). According to the distribution characteristics of the AE sources, many high-energy events were concentrated near the upper discontinuity, while some low-energy events gathered near the bottom discontinuity (Fig. 4c). Due to significant attenuation effect of macroscopic cracks in the rock parting on AE energy, a large number of fracture events were not located and recorded, resulting in a lower frequency. For sample #4, both the upper and bottom discontinuities produced obvious slip, and the coal blocks and rock parting did not generate significant fractures (Fig. 3d). A large number of low-energy sources were concentrated near the two discontinuities, indicating slip without obvious fractures (Fig. 4d).

**Figure 5:**
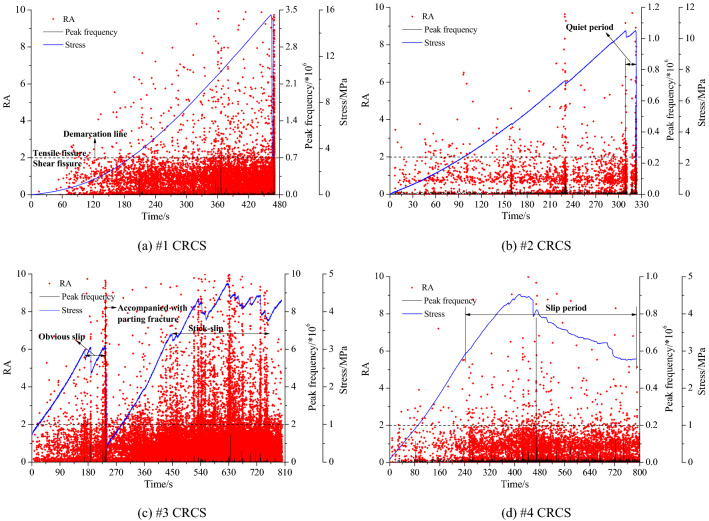
Relationships between RA, peak frequency, and stress during the process of slip, fracture, and instability.

A large number of microcracks will be produced accompanied by deformation, slip, and fracture until the compound samples reach instability. Knowing the type and evolution of these microcracks is helpful to clearly understand the microscopic mechanism of instability and failure, especially for slip characteristics. Figure 5 shows the RA value (the ratio of impact rise time to AE amplitude) and the peak frequency during the complete process of slip, fracture, and instability of the four samples.

A greater RA value indicates tensile cracks, whereas a lower RA value indicates shear cracks. During the instability and failure process of sample #1, a large number of tensile cracks were generated and there were no obvious shear cracks at the initial stage of loading (Fig. 5a). A larger number of tensile and shear cracks appeared with loading and the final instability was characterized by tensile failure of the lower coal. Meanwhile, the peak frequency of AE reached a maximum value. As shown in Fig. 5b, a large number of shear cracks generated at the initial stage of loading, and the entire slip and instability process was mainly characterized by shear failure, with few tensile cracks. Each stress drop corresponded to a peak frequency of AE generated by tensile cracks. Before instability, a quiet period was experienced, and the final failure was accompanied by obvious shear and tensile cracking. For sample #3 (Fig. 5c), shear fracture predominately appeared at the initial stage of loading, accompanied by several tensile cracks. Each stress drop corresponded to a peak frequency of AE, and the tensile cracks increased significantly, which fully demonstrated the stick–slip of the two discontinuities. Each slip produced a significant stress drop, and a large number of shear cracks were formed, accompanied by some tensile cracks. As shown in Fig. 5d, the complete slip and instability process of sample #4 was dominated by shear cracking, with fewer tensile cracks. A microslip appeared during the slip stage, which was characterized by a nonobvious stress drop. A large slip producing a significant stress drop was observed, which corresponded to a peak frequency of AE.

In summary, each slip of the discontinuities generated both shear and tensile cracks, but the former was dominant. In addition, RA = 2 can be used as a basis for discriminating between shear and tensile cracks.

#### Deformation and AE characteristics

**Figure 6:**
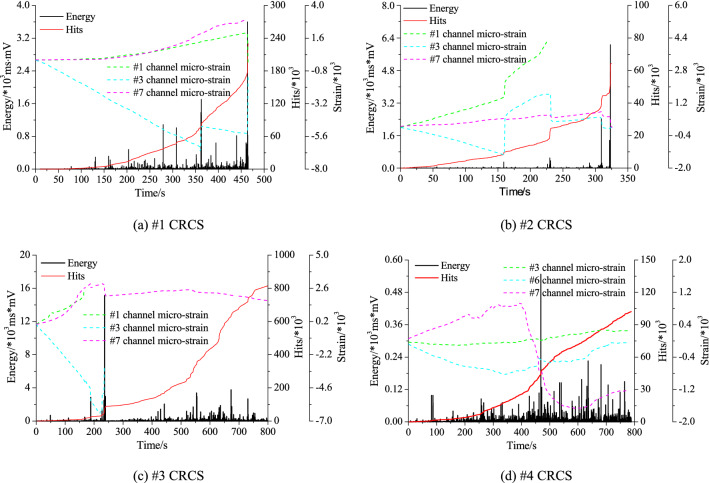
Variations in AE energy, hits, and strain of coal and rock paring sublayers during the entire loading process.

Strain gauges and AE sensors were used to monitor and record the whole process of slip and instability of the samples. Figure 6 shows the variations in AE energy, hits, and strain of the coal and rock parting sublayers during the whole loading process.

As shown in Fig. 6a, the AE energy was low (below 2 × 10^3^ ms·mV) before instability, demonstrating slight fracture and slip. After occurrence of final instability, the energy instantly reached a peak equal to 3.598 × 10^3^ ms·mV. During the entire loading process, the AE hits continued to rise and suddenly reached a peak once instability was reached. The circumferential strain of gauge #1 on the upper coal and gauge #7 on the bottom coal gradually increased with loading, while the axial strain of gauge #3 on the upper coal increased sharply. Due to macroscopic fracture (*t* = 364 s), the strain of the #3 gauge dropped and slightly increased with continuous loading, and it suddenly dropped to the minimum value again when final instability occurred. As shown in Fig. 6b, the AE energy of the #2 sample was extremely low before instability, in which the first two energy peaks were only approximately 0.5 × 10^3^ ms·mV. Moreover, three slips produced three energy peaks (*t* = 160 s; *t* = 230 s; and *t* = 310 s) corresponding to the sudden increase in AE hits, and each slip caused a strain mutation of the coal blocks. The energy at final instability reached 6.069 × 10^3^ ms·mV, which was significantly higher than that of sample #1, while the axial stress of the former was obviously lower than that of the latter. The overall level of AE energy was high with an extreme value of 15.128 × 10^3^ ms·mV (Fig. 6c), which was generated by fracture of coal and rock parting during loading, whereas the stress peak (approximately 4.75 MPa) was much lower than that of sample #1. The #1 and #3 strain gauges failed due to macroscopic fracture of the upper coal. Each slip corresponded to an energy peak and a sudden increase in hits. As shown in Fig. 6d, the AE energy level was lowest, and the stress peak was almost equivalent to that of sample #3 when final instability was reached. The AE hits continued to increase during loading, and each slip corresponded to an energy peak. In summary, the AE effects were characterized by low energies and hits due to continuously stable slip without obvious fracturing of the coal and rock parting.

The results can be summarized as follows. (1) The peak stress was the highest for pure fracture and instability without obvious slip, and the energy was higher with gradual growth and development of cracks inside the coal and rock parting. In particular, the energy reached a peak when instability occurred. (2) The slip and instability were usually accompanied by the growth and propagation of cracks, and the AE energy was the highest. The instability mode was characterized by low stress and a high energy. (3) For continuously stable slip with fewer cracks, both the peak stress and the released energy were smaller. Each stick–slip corresponded to an AE energy peak.

### Precursory parameters associated with slip and instability

#### The *b* value

As a function for descripting the scale of crack propagation, the *b* value of AE events can be used to track the evolution of fractures in coal and rock mass^47^. Gutenberg and Richter put forward the *G*–*R* relationship between magnitude and frequency of seismicity^48^:

1 $$\lg N = a-bm$$

where *m* is the magnitude, and *N* is the event count with a magnitude within *Δm*; *a* and *b* are constants, and the *b* value is usually determined by the maximum likelihood method^49^:

2 $$b = \frac{{\log_{10}^{e} }}{{\overline{M} - \left( {M_{c} - \frac{\Delta M}{2}} \right)}}$$

where ${\overline{{M}}}$ is the mean magnitude, *M*_*c*_ is the magnitude of completeness, and *ΔM* is the distribution width of the magnitude. ${\text{M}}_{{\text{c}}} {-}\frac{{\Delta {\text{M}}}}{{2}}$ can be regarded as the initial magnitude.

**Figure 7:**
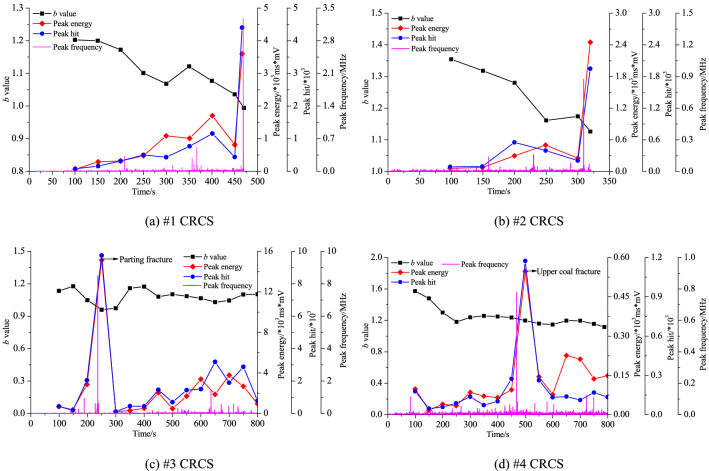
Relationships between the *b* value, peak energy, peak hits, and peak frequency of AE during entire loading process.

Figure 7 shows the relationships between the *b* value, peak energy, peak hits, and peak frequency of AE during the slip and instability of the four compound samples.

As shown in Fig. 7a, the AE signals did not appear at the initial stage of loading. The *b* value exhibited a continuously decreasing trend and reached a minimum value upon final instability. The peak energy and hits showed a continuously rising trend and dropped prior to final instability, and then suddenly increased to maximum values. Each microcrack corresponded to a high peak frequency (especially *t* = 350 s), and the *b* value increased, indicating the formation of a large number of microcracks. At the final instability stage, the peak frequency reached its maximum, which was mainly due to macrofracturing of the coal and rock parting. As shown in Fig. 7b, both the peak energy and hits were small during 0–100 s and began to rise with loading, accompanied by a rapid decrease in the *b* value; before final instability, both decreased to a low level, and the *b* value increased slightly, indicating the slip of the discontinuities. When final instability occurred, the *b* value dropped, followed by sudden increase in the peak energy and hits. During 0–250 s, the peak energy, hits, and frequency suddenly increased to their maximum values due to rock parting fracture (Fig. 7c), accompanied by a sudden drop in the *b* value, and then the peak energy and hits dropped. During slip, the peak energy and hits fluctuated frequently, the overall level of the peak frequency was lower, and especially the *b* value basically remained stable, verifying the macroscopic slip of the discontinuities. The higher levels of peak energy and hits indicated a large number of microcracks during slip. The peak energy, hits, and frequency were small during the entire loading process, except for a peak generated by the upper coal fracture (Fig. 7d). At the initial loading stage, the *b* value was higher and then gradually decreased, indicating the gradual growth, propagation, convergence, and coalescence of microcracks. During steady slip, the *b* value was relatively stable.

In summary, for samples #1 and #2 characterized by fracture instability without obvious slip, the peak energy and hits of AE first decreased and then suddenly increased, accompanied by a sudden drop in *b* value; thus, the precursory effect was obvious. For instability with obvious slip (samples #3 and #4), the peak energy and hits fluctuated periodically with slip, and the *b* value basically remained stable. Each slip corresponded to a peak frequency. Therefore, precursors were difficult to identify. In addition, the peak frequency can reveal the final instability mode: a high frequency corresponds to fracture, whereas a low frequency corresponds to slip.

#### FTA evolution

**Figure 8:**
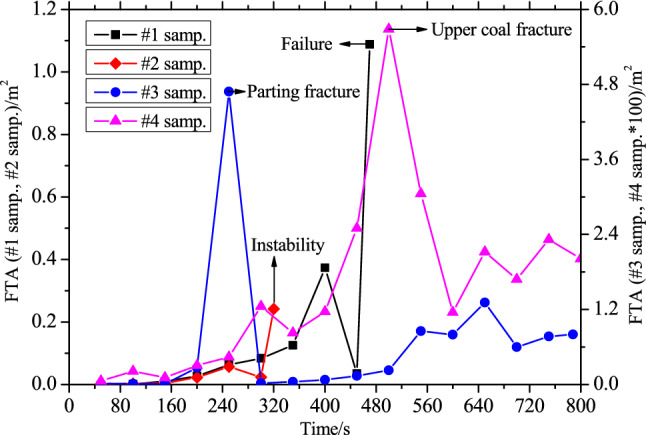
Evolutions of FTA of the four compound samples during the entire loading process.

Strong AE events tend to occur in fault zones, and a large amount of energy accumulated in the seismogenic process will release during crack growth. This is because the new cracks initiate and grow from pre-existing defects in rocks subjected to the compression stress state. Therefore, more pre-existing defects in the fault zone lead to a greater possibility of strong AE. Theoretically, before high-energy release, the FTA commonly presents an abnormally high value, indicating that AE will obviously be enhanced. Figure 8 shows the FTA evolution of the four compound samples during fracture, slip, and instability.

From Fig. 8, the FTA of sample #1 continued to rise and then dropped prior to instability, and it suddenly increased to a maximum during final instability. The FTA variation of sample #2 was similar to that of sample #1. For sample #3, the FTA first showed a gradual increasing trend (except for reaching a maximum upon rock parting fracture) and dropped prior to instability, and it then slightly rose until reaching final instability, which was different from the samples #1 and sample #2. For sample #4, the FTA reached a maximum upon upper coal fracture and then dropped. At the later slip stage, the FTA periodically fluctuated, which was accompanied by the formation of microcracks. In addition, the order of the FTA values was sample #1 > sample #2 > sample #4 > sample #3, thus demonstrating that the FTA of the fracture and instability mode was much larger than that of the slip and instability mode.

In summary, prior to fracture and slip instability, microcracks coalesced and formed macrofractures, simultaneously initiating new microcracks, which caused a sudden drop in or low value of FTA. Thus, the sudden drop in FTA can be regarded as a precursor for warning fracture or slip instability.

## Field investigation

### Overview of working face and setting of MS system

Bifurcation is common in coal seam #3 in the Shandong mining area, China. The failure and instability characteristics of the coal and rock parting compound structure were analyzed using MS data during the advancing of the 8301 working face with coal seam bifurcation in a mine.

**Figure 9:**
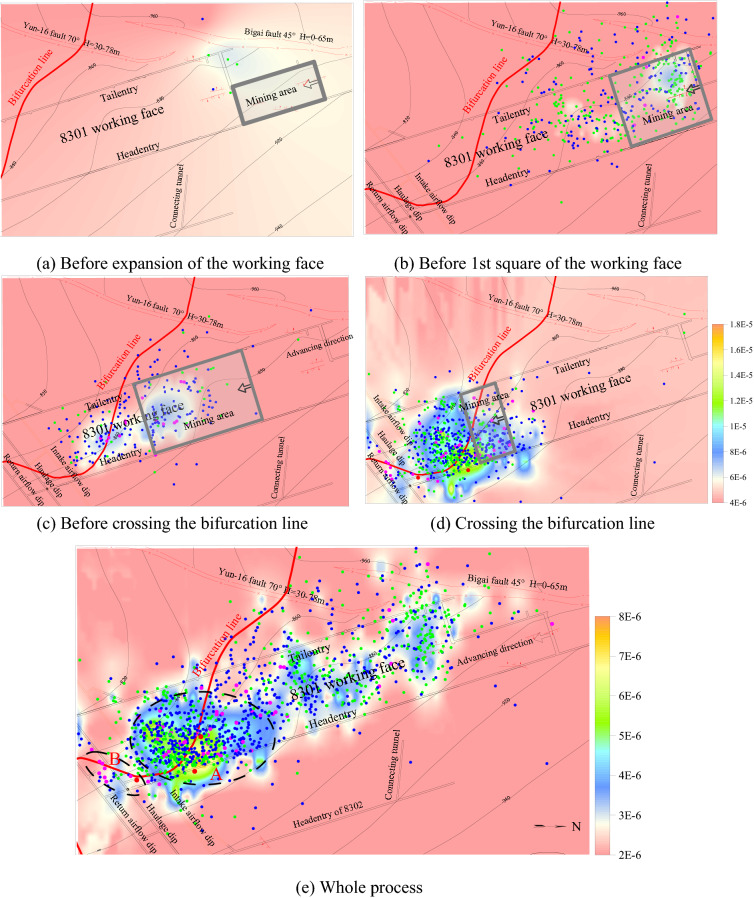
Plane sketch of the 8301 working face, kernel density distribution of MS events, and location of the events with different energy levels. Note: the legend in (**a**)–(**d**) is same; and (**e**) the energy levels are represented by different colors of source events: green: < 10^3^ J; blue: 10^3^–5 × 10^3^ J; magenta: 5 × 10^3^–10^4^ J; and red: 10^4^–5 × 10^4^ J).

The 8301 working face is the first mining face in the mining area #8, with a buried depth of 875.7–961.2 m. The dip angle of the coal seam is 0°–11°, and there is a layer of medium sandstone with a thickness of 15.1 m in the immediate roof above the coal seam, as well as a layer of fine sandstone with a thickness of 9.1 m in the primary roof. Furthermore, there is a layer of fine sandstone with a thickness of 8.4 m in the primary floor, and the thickness of other adjacent strata is small. According to identification results, the coal seam, roof, and floor are characterized by a weak rockburst tendency. The faults in the working-face area are the FX48 fault and Yun 16 fault; fortunately, both drops are small within the working face with a minor impact on the mining. A bifurcation line is distributed near the stoping line of the working face, the thickness of the lower coal layer is 4.2–6.0 m in the bifurcation area, and the thickness of the rock parting is 0–6.9 m. The rock parting is mainly composed of mudstone or carbonaceous mudstone and siltstone, or fine sandstone in some sections. Considering the risk of rockburst, pressure relief of the coal and rock has been carried out before and during the advancing of the working face. A plane sketch of the working face is shown in Fig. 9.

In addition, an MS monitoring system was installed in the mine with more than 30 geophones, of which 5 geophones were distributed near the 8301 working face; the locations are given in Table 2.

**Table 2:** Three-dimensional coordinates of five geophones.

Geophone no.	x/m	y/m	z/m
T3	20,401,540	3,909,734	− 862
S10	20,401,333	3,909,338	− 827
T11	20,401,277	3,909,711	− 843
S14	20,401,976	3,909,822	− 916
T12	20,401,312	3,909,598	− 832

### Distribution and signal characteristics of MS source

Figure 9 shows the kernel density distribution of MS events and their location with different energy levels based on the MS data during the advancing of the working face.

According to the characteristics of MS events during different advancing stages (Fig. 9a–d), only few low energy MS events occurred before expansion of the working face, while the number and energy of MS events significantly increased after expansion of the working face. After the working face advanced to the location of 1st square, the number of MS events decreased, but the number of high-energy events significantly increased, with an obvious concentration of MS events in the bifurcation area. When the working face advanced crossing the bifurcation line, the number and energy of MS events significantly increased again, and the events were obviously concentrated near the bifurcation line. According to the kernel density distribution and location of MS events during the whole advancing process (Fig. 9e), high values of kernel density was mainly concentrated in three regions: the expansion of the working face, 1^st^ square of the working face and near the bifurcation line, and there are also sporadic high value areas near the fault area. It indicated that the expansion of the working face, square of the working face, coal seam bifurcation, and fault structure promoted the fracture of the coal and rock. It was obvious that the kernel density of MS events near the bifurcation line (area A) was significantly higher than that in other areas, particularly in the area adjacent to the bifurcation line and headentry, where the kernel density reached a maximum, which indicated that the coal and rock in the coal seam bifurcation area obviously fractured under the abutment pressure of the working face, with many MS events. Further, the fracture events in the area adjacent to the bifurcation line and headentry were the most affected by the superposition of roadway intersections and coal seam bifurcation. According to the location of the MS events, their energy levels were generally small in the areas of working face expansion, squaring, and fault structure, indicating that only a small-scale fracture of coal and rock occurred in these areas. In contrast, the energy of the events was high near the bifurcation line, especially in the intersection areas between the bifurcation line and the roadways (area B and area A nearby the headentry), and the high-energy events were relatively concentrated. It can be inferred that the coal and rock in the bifurcation area generated macrofracture/slip.

**Figure 10:**
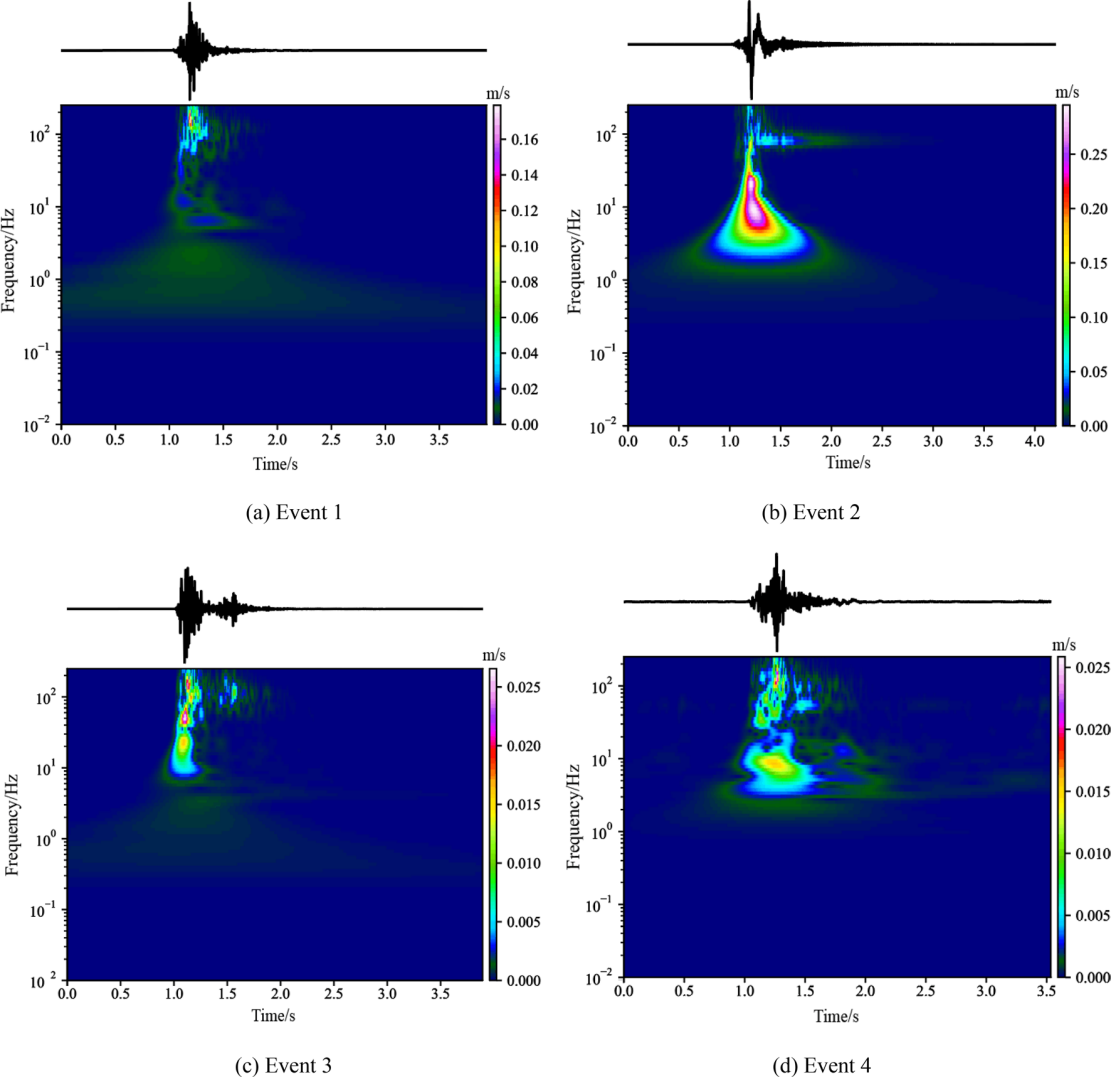
Wave velocity and spectrum distribution of MS waveforms.

The wave velocity and spectrum distribution of 4 MS events located near the bifurcation line are given in Fig. 10 to roughly analyze the fracture type of the coal/rock in the bifurcation area. Event 1 was located outside the bifurcation area, event 2 was close to the bifurcation line, and events 3 and 4 were located inside the bifurcation area.

According to Fig. 10, both the duration and velocity of event 1 were significantly less than that of the other three events, and the dominant frequency was mainly concentrated in the high frequency region (> 100 Hz). Compared with the other events, the vibration velocity of event 2 was the highest, and the dominant frequency was mainly concentrated in the low-frequency region (< 40 Hz). The vibration velocities of events 3 and 4 were similar, and both of the dominant frequencies were widely distributed, including obvious low-frequency components (< 60 Hz) and high-frequency components (> 100 Hz). According to the distribution characteristics of the main frequency, it can be inferred that the fracturing of the coal/rock mainly occurred outside the bifurcation line, while there were obvious shear slip in the areas near the bifurcation line and inside the bifurcation area. Shear slip was accompanied by obvious fracturing of the coal/rock in the bifurcation area.

### Multi-parameter evolution of MS

**Figure 11:**
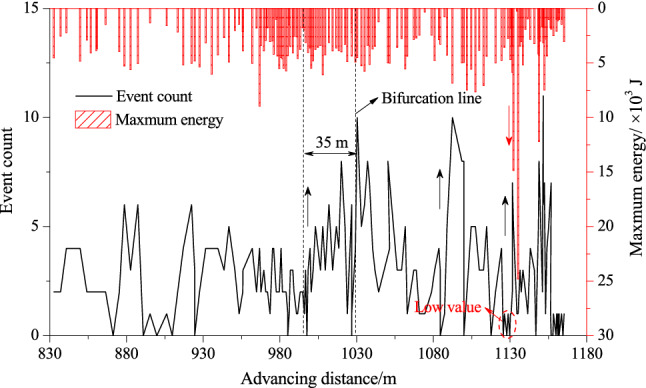
Variations in MS parameters during the working face advancing near the coal seam bifurcation line.

Based on the MS data, the statistics of the event count and the maximum energy during the working face advancing near the bifurcation line are shown in Fig. 11.

Affected by the abutment pressure of the working face, the MS event count gradually increased from 35 m away from the bifurcation line until the bifurcation area, while an increase in the maximum energy was not obvious (Fig. 11), inferring that fracturing of the coal and rock near the coal seam bifurcation started to occur at this stage, but it was mainly of a small scale. A high MS event count appeared frequently after the coal seam bifurcation line was exposed, which then gradually decreased, whereas the MS maximum energy level increased on a small scale, indicating the deformation and fracturing of the coal and rock in the bifurcation area, which was similar to the fracture characteristics of sample #3 in the laboratory test. It is worth noting that the working face was close to the adjacent area of bifurcation line and the headentry at this stage, which shows that large-scale fracture/shear slip of the coal and rock compound structure occurred under the superposition of mining disturbance and roadway intersections. In addition, consistent with the experimental results, there was an obvious energy storage stage with a low event count and a maximum energy before large energy level events.

**Figure 12:**
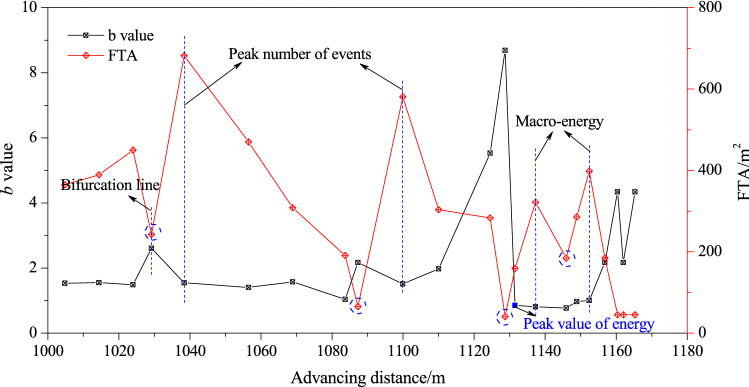
Variations of *b* value and FTA during the working face advancing in the coal seam bifurcation area.

According to the variations of *b* value during the working face advancing in the bifurcation area (Fig. 12), the *b* value always increased first and then decreased before the significant increase in event count, particularly before the occurrence of high-energy events; the *b* value first increased to a maximum value and then rapidly decreased to a minimum value, which may be related to the massive initiation and macroexpansion of microcracks before the macrofracture/slip of the composite structure of coal and rock.

Correspondingly, low FTA values of MS events always appeared before a significant increase in the MS event count or the occurrence of high-energy events, and then FTA increased rapidly accompanied by a significant increase in the MS event count or the occurrence of high-energy events, indicating that there was an obvious energy storage process before the fracture/sliding of the composite structure of coal and rock in the bifurcation area, which was consistent with the experimental results.

## Conclusions

1. Each slip of the discontinuities produced shear and tensile cracks, but the former was dominant. RA = 2 can be used as a basis for discriminating between shear and tensile cracks.
2. For the instability mode, which was characterized by low peak stress, high energy release, and a stable *b* value of AE, each slip corresponded to a peak frequency. The high frequency corresponded to a fracture, and a low frequency corresponded to a slip.
3. Prior to fracture and slip instability, microcracks grew, coalesced, and formed macrofracture. Meanwhile, the initiation of new microcracks was delayed, which caused a sudden drop in or a low value of FTA. The sudden drop in FTA can be regarded as a precursor for warning fracture or slip instability of a discontinuity.
4. Field investigations showed that the MS events near the coal seam bifurcation line were obviously concentrated. Especially in the intersection areas between the bifurcation line and the roadway, many high-energy events occurred. There were mainly high-frequency fracture events distributed from the bifurcation line, and obvious low-frequency shear fracture occurred near the coal seam bifurcation line and in the bifurcation area. Moreover, the evolution law of the *b* value and FTA, as well as the precursory effects of the macrofracture/slip of the compound structure, verified the conclusions of the experimental tests.
5. The research can realize the early warning of failure and instability of coal/rock in bifurcation area, by analyzing the RA, *b* value, FTA, energy release, and events location based on the MS data during advancing of working face, which can decrease the danger of coal/rock dynamic disaster in mines.

## Data Availability

The datasets used and analysed during the current study available from the corresponding author on reasonable request.
